# Using SpO_2_ Recovery Index after a 6-Minute Walk Test to Predict Respiratory-Related Events in Hospitalized Patients with Interstitial Pneumonia

**DOI:** 10.1038/s41598-019-51818-1

**Published:** 2019-10-23

**Authors:** Yasunari Sakai, Shuhei Yamamoto, Miho Hoshina, Shohei Kawachi, Takashi Ichiyama, Masayuki Hanaoka

**Affiliations:** 1 Rehabilitation, Shinshu University Hospital, Matsumoto, Japan; 2 Respiratory Center, Shinshu University Hospital, Matsumoto, Japan

**Keywords:** Diseases, Risk factors

## Abstract

Although the prognostic factors of interstitial pneumonia (IP) patients have been reported, IP has poor prognosis. Hospitalized patients with IP have severely impaired pulmonary diffusion capacity and prominent desaturation. We hypothesized that determining oxygen saturation recovery (SpO_2_ recovery index) after the 6-minute walk test (6MWT) can provide additional prognostic information regarding rehospitalization for respiratory-related events. We evaluated 73 IP patients at our hospital for demographic characteristics, pulmonary function tests and 6MWT. The Kaplan–Meier method was used to estimate rehospitalisation for respiratory-related events using SpO_2_ recovery index. Cox regression analysis revealed a relationship between SpO_2_ recovery index and rehospitalisation. The optimum cutoff value of SpO_2_ recovery index was 4% (sensitivity, 71.4%; specificity, 79.2%). SpO_2_ recovery index was most closely related to pulmonary diffusion capacity (*r* = 0.684, *P* < 0.001). In a multivariable model, it was the strongest independent predictor of rehospitalisation for respiratory-related events (hazard ratio, 0.3; 95% confidence interval, 0.10–0.90; *P* = 0.032). In this study, we estimated pulmonary diffusion capacity using SpO_2_ recovery index values obtained from 6MWT. A SpO_2_ recovery index of <4% can be useful in predicting rehospitalisation for respiratory-related events.

## Introduction

Central pathophysiological features of interstitial pneumonia (IP) include impaired degradation of pulmonary diffusion capacity due to pulmonary interstitium and capillary bed disorders, ventilation perfusion ratio mismatch, and worsening gas exchange with exercise^1^. Dyspnea during exercise reduces exercise tolerance, ability to perform daily activities, and health-related quality of life^2^. Although prognostic factors for IP have been reported, IP has poor prognosis^3–8^, including reduced pulmonary diffusion capacity and hypoxemia are poor prognostic factors in patients with IP^3,4^. Also, measuring partial oxygen pressure (PaO_2_) during exercise predicts survival in patients with IP^5^. Moreover, desaturation during the 6-minute walk test (6MWT) is a prognostic factor for patients with IP in stable conditions^6^. Exercise-induced hypoxemia (EIH) of patients with IP is related to the pulmonary diffusion function, which is also one of the prognostic factors^6–8^.

A simple test to evaluate desaturation is used clinically in 6MWT^9,10^. Heart rate recovery (HRR) obtained from 6MWT has drawn attention as a prognostic factor in patients with IP and in those with chronic obstructive pulmonary disease and heart failure^11,12^. Heart rate recovery 1 (HRR1), obtained by subtracting heart rate (HR) after 1-min walk, from the rate at 6MWT end, showed the strongest significance for mortality^13,14^. Considering the physiologic response after exercise as a prognostic indicator is essential. Hospitalized patients with IP have severely impaired pulmonary diffusion capacity and prominent desaturation; changes in HR after exercise and desaturation evaluation are important. Studies on the relationship between mortality and clinical indicators during and after exercise included stable IP outpatients^13,14^. To our knowledge, no study has evaluated hospitalized IP-patient mortality. HR during and HRR after exercise and all-cause mortality are reported, but readmission/mortality predictive factors have not been considered, restricting acute IP exacerbation and respiratory failure^15–17^. During rehabilitation of hospitalised patients with IP with an unstable respiratory state, pulmonary diffusion capacity should be considered. Determining the breathing pattern, exercise oxygen saturation, post-exercise oxygen saturation recovery and HR changes is crucial because rehospitalisation for acute IP exacerbation and respiratory failure accounts for a majority of the deaths in this disease; therefore, the results of such evaluations constitute useful information for risk management. Therefore, we examined whether oxygen saturation after 6MWT in hospitalized patients with IP reflected pulmonary diffusion capacity. Oxygen saturation recovery after 6MWT was hypothesized to provide additional prognostic information regarding rehospitalization for respiratory-related events in these patients.

## Materials and Methods

### Ethical approval

The study protocol was approved by the ethics committee of Shinshu University (No. 3732) and conducted according to the Declaration of Helsinki (latest version). Written informed consent was obtained from all participants after detailed explanation of the study protocol.

### Patient selection

Patients with IP who were hospitalized at Shinshu University Hospital in Japan during January 2015-July 2018 were selected. IP was diagnosed based on the American Thoracic Society/Infectious Diseases Society of America guidelines^18^.

Patients with underlying gait disturbance due to cerebral infarction/collagen vascular disease/occupational exposure/high-flow oxygen and those with recognized cognitive decline (23 points > Mini-Mental State Examination)^19^ were excluded.

### Procedure

This study is a cohort study. Demographic characteristics (age, gender, body mass index; BMI), diagnosis (idiopayhic pulmonary fibrosis, acute interstitial pneumonia, combined pulmonary fibrosis and emphysema), treatment (prednisolone, supplemental O_2_ flow), pulmonary function tests, 6MWT and laboratory data on admission (krebs von den lungen-6, PaO_2_/FIO_2_ ratio) were collected upon hospital discharge. 6MWT was performed upon hospital discharge and indicators were collected. Pulmonary function tests, including spirometry and pulmonary diffusion capacity for carbon monoxide (DLco), and changes in nitrogen levels (ΔN_2_) were measured as previously described^20^. Forced vital capacity (FVC) and DLco were assessed according to the American Thoracic Society/European Respiratory Society (ATS/ERS) criteria; their results were reported as predicted value percentages^21–23^. Pulmonary function tests using CHESTAC-8900 (CHEST, Tokyo, Japan) were conducted. Pulmonary hypertension (PH) was diagnosed based on the estimated pulmonary artery systolic pressure calculated using the simple Bernoulli equation [estimated pulmonary artery systolic pressure = 4 × (3 tricuspid regurgitation velocity)^2^ + estimated right atrial pressure] with reference to trans tricuspid pressure gradient determined using echocardiography^24^.

### 6MWT

Walking path and criteria for terminating measurement was set by the ATS guidelines^25^. 6MWT protocol was designed to accurately assess oxygen desaturation and to provide a clinically useful oxygen titration. All patients’ baseline HR and saturation of percutaneous oxygen (SpO_2_) using pulse oximetry (Pulsox-M, Teijin Ltd, Tokyo, Japan) were measured. For patient safety, the test was discontinued on noting unbearable chest pain/shortness of breath/new arrhythmia development. To accurately assess SpO_2_, the respiratory therapist confirmed an acceptable pulse oximeter signal and pulsing of oximeter bar indicating synchrony between HR before beginning all tests. Patients walked on a level surface with gentle vocal encouragement using set phrases every 1 min. SpO_2_ and HR were continuously measured during 6MWT, and in all patients modified Borg scale at 6MWT end and distance were measured. Change in SpO_2_ (ΔSpO_2_) was the difference between a subject’s resting SpO_2_ and the lowest SpO_2_ during 6MWT (resting – lowest SpO_2_). SpO_2_ recovery index was the difference between a subject’s SpO_2_ at 6MWT end (<6 min if the test was terminated for a low SpO_2_) and 1 min into recovery and dividing by the resting SpO_2_: [(SpO_2_ 1 min into recovery after 6MWT − lowest SpO_2_)/resting SpO_2_] × 100.

### End point

Subjects were followed-up with after 12 months; any major respiratory-related events requiring hospitalization were recorded.

### Statistical analysis

The subjects were classified into two groups: not-rehospitalization (event free) and rehospitalization due to respiratory-related events groups. Categorical and continuous data were compared using the χ^2^ and Mann-Whitney *U* tests, respectively. Correlations between quantitative variables of the lowest SpO_2_, ΔSpO_2_, SpO_2_ at 1 min after 6MWT, distance, SpO_2_ recovery index, and %DLco in the entire population were evaluated using the bivariate Pearson test. Rehospitalization SpO_2_ recovery index validity was determined considering rehospitalization and SpO_2_ recovery index as objective and explanatory variables, respectively. The receiver operating characteristic (ROC) curve and area under the ROC curve (AUC) were determined. An optimal cutoff point maximizing sensitivity and specificity for SpO_2_ recovery index was determined from sensitivity and specificity curve intersection^26^.

The product-limit method was used to derive rehospitalization for respiratory-related events; Kaplan-Meier curves were used to display rehospitalization for the study sample stratified by SpO_2_ recovery index. For multivariable analysis of rehospitalization due to respiratory-related events to develop the most parsimonious model, candidate variables included those with a *P* < 0.05 on bivariate analysis. Cox regression analysis examined the relationship between SpO_2_ recovery index and rehospitalization; adjusting for demographic characteristics; PH diagnosis and complication; treatment; and physiologic, 6MWT, and laboratory data. Overfitting was avoided by reducing potential confounding factors including SpO_2_ recovery index to a single composite characteristic by applying a propensity score.

Analyses were performed using SPSS 24.0 software (IBM Japan, Tokyo, Japan). Descriptive statistics [mean ± standard deviation (SD)] were used. All tests assumed unequal variances; *P* < 0.05 indicated statistical significance.

## Results

### Follow-up on rehospitalization

No patient was lost to follow-up during the subsequent 12 months. Fourteen respiratory-related events (10 IP acute exacerbations and 4 respiratory failures) occurred during the tracking period (event rate = 19.2%).

### Participant characteristics

Table 1 shows clinical characteristics of participants in the two groups. There were no between-group significant differences in age, gender percentage, BMI, diagnosis, prednisolone use, forced expiratory volume (FEV_1.0_), ΔN_2_, resting SpO_2_, ΔSpO_2_, modified Borg scale at 6MWT end, and clinical laboratory data.

**Table 1:** Clinical Characteristics.

Variable	Not readmission (n = 59)	Readmission (n = 14)	*P-value*
Demographics			
Age (years)	68.9 (7.7)	66.7 (10.4)	0.468
Men/women, n (%)	37 (63)/22 (37)	9 (64)/5 (36)	0.42
BMI (kg/m^2^)	20.5 (3.1)	19.6 (4.4)	0.139
Diagnosis			
IPF, n (%)	32 (54)	8 (57)	0.664
AIP, n (%)	19 (32)	4 (29)	0.657
CPFE, n (%)	8 (14)	2 (14)	0.382
Complication of PH, n (%)	12 (20)	4 (28)	0.036
Treatment			
Use PSL (mg/day)	29.7 (17.4)	30.7 (11.9)	0.993
O_2_ flow (L/min)	1.6 (1.3)	2.8 (1.5)	0.016
Physiologic			
FVC predicted (%)	68.0 (19.2)	48.1 (10.6)	*P* < 0.001
FEV_1.0_ (%)	75.3 (14.8)	69.6 (14.7)	0.17
DLco predicted (%)	46.5 (14.8)	32.6 (16.5)	0.006
Δ N_2_ (%)	4.1 (1.8)	4.9 (1.6)	0.089
6MWT data			
Resting SpO_2_ (%)	95.0 (1.6)	94.1 (1.9)	0.187
Distance (m)	331.7 (134.6)	227.1 (148.9)	0.016
HRR1 (beat)	18.6 (8.0)	13.9 (5.6)	0.026
Lowest SpO_2_ (%)	85.3 (4.8)	82.9 (4.3)	0.028
Δ SpO_2_ (%)	9.5 (4.9)	11.2 (3.8)	0.072
SpO_2_ 1 minute (%)	90.1 (5.0)	86.6 (4.6)	0.011
SpO_2_ recovery index (%)	5.1 (1.8)	3.8 (1.8)	0.026
Modifide Borg Scale	3.5 (1.5)	4.6 (2.2)	0.065
Laboratory data			
KL-6 (U/ml)	1308 (194.0)	1528.5 (1017.0)	0.674
P/F ratio	303.0 (71.3)	261.6 (97.6)	0.12

P-values for comparison between groups stratified on readmission. Data are counts (percentages), mean (SD). Definition of abbreviations: BMI indicates body mass index; IPF, Idiopayhic pulmonary fibrosis; AIP, Acute interstitial pneumonia; CPFE, Combined pulmonary fibrosis and emphysema; PH, Pulmonary hypertension; PSL, Prednisolone; O_2_ flow, Supplemental O_2_ flow; HRR1, HR at the end of 6MWT minus HR after 1 minute at the end of 6MWT; Δ SpO_2_, SpO_2_ at the end of the 6MWT minus SpO_2_ at baseline; SpO_2_ 1 minute, SpO_2_ 1 min into recovery after 6MWT; SpO_2_ recovery index; {(SpO_2_ 1 minute in to recovery after 6MWT minus lowest SpO_2_)/resting SpO_2_} × 100; KL-6, Krebs von den Lungen-6; P/F ratio, PaO_2_/FIO_2_ ratio.

### Correlation analysis

**Figure 1:**
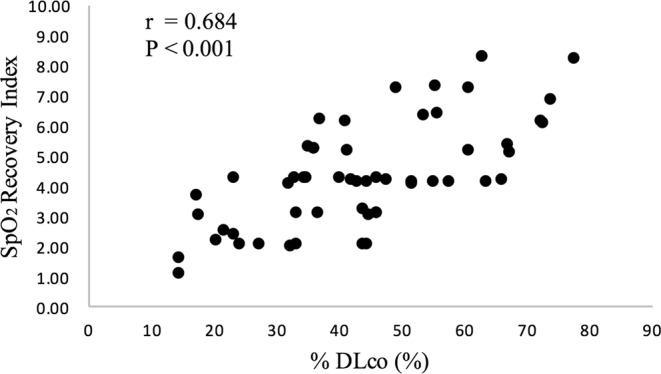
Correlations between %DLco, SpO_2_ recovery index. %DLco, DLco predicted; SpO_2_ recovery index, {(SpO_2_ 1 minute in to recovery after 6MWT minus lowest SpO_2_)/resting SpO_2_} × 100.

Lowest SpO_2_, ΔSpO_2_, SpO_2_ 1 min after 6MWT, distance, and SpO_2_ recovery index significantly correlated with %DLco with moderate correlation coefficients. SpO_2_ recovery index obtained from 6MWT had the highest correlation coefficient (*r* = 0.684, *P* < 0.001; Fig. 1) including other evaluations.

### ROC curves and related rehospitalization analysis

**Figure 2:**
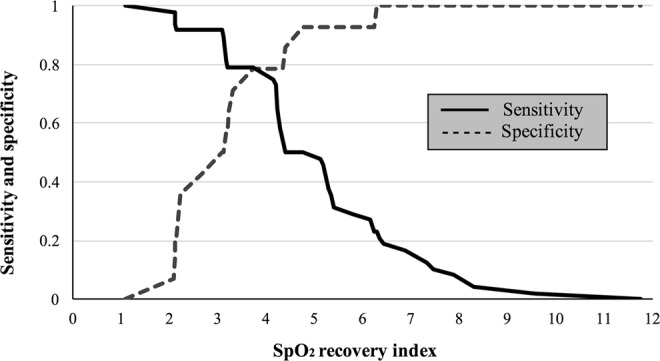
ROC curves to predict readmission of respiratory related events by SpO_2_ recovery index. Cutoff 4%, AUC 0.75 (P = 0.005), sensitivity 71.4%, specificity 79.2%, positive likelihood ratio 3.43.

Figure 2 shows the ROC curve of SpO_2_ recovery index to deduce rehospitalization of respiratory-related events. AUC showing the usefulness of SpO_2_ recovery index usefulness was significantly higher at 0.75 (standard error = 0.07; *P* = 0.005). The optimum cutoff value of SpO_2_ recovery index was 4% (sensitivity = 71.4%; specificity = 79.2%). The positive likelihood ratio of in the cutoff of SpO_2_ recovery index was 3.43.

### Kaplan-Meier curves

**Figure 3:**
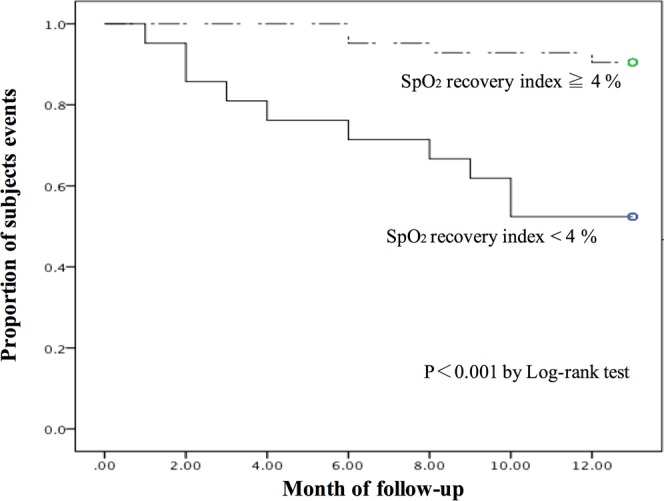
Cumulative event-free probability of patients with IP according to SpO_2_ recovery index estimated by the Kaplan-Meier method. Stratified according to whether SpO_2_ recovery index ≧4% or SpO_2_ recovery index <4%.

Figure 3 shows Kaplan-Meier analyses of rehospitalization for the study sample stratified by SpO_2_ recovery index (≥4% vs <4%). In time-to-event survival analysis, SpO_2_ recovery index was significantly associated with respiratory-related events for patients with IP (*P* < 0.001).

### Multivariate Cox regression analysis

Table 2 shows a Cox proportional hazard model. In multivariable analyses using the propensity score, SpO_2_ recovery index [hazard ratio (HzR) = 0.3; 95% confidence interval (CI) = 0.10–0.90; *P* = 0.032] and HHR1 (HzR = 0.91; 95% CI = 0.82–0.99; *P* = 0.045) were significantly associated with respiratory-related events. Among them, SpO_2_ recovery index was the most associated with respiratory-related events.

**Table 2:** Cox Proportional Hazard Respiratory Related Events Analysis.

Variable	Univariable analysis		Multivariate analysis					
			Model 1		Model 2		Model 3	
	HR (95% CI)	*P-value*	HR (95% CI)	*P-value*	HR (95% CI)	*P-value*	HR (95% CI)	*P-value*
SpO_2_ recovery index (%)	0.45 (0.29–0.72)	0.001	0.30 (0.10–0.90)	0.032	—	—	—	—
HRR1 (beat)	0.84 (0.90–0.97)	0.032	—	—	0.91 (0.82–0.99)	0.045	—	—
6MWT distance (m)	0.99 (0.98–0.99)	0.017	—	—	—	—	1.00 (0.99–1.01)	0.810
Demographics								
Age (years)	0.97 (0.91–1.03)	0.307	ND	ND	ND	ND	ND	ND
Sex (men vs women)	1.01 (0.34–3.34)	0.980	ND	ND	ND	ND	ND	ND
BMI (kg/m^2^)	0.91 (0.75–1.10)	0.358	ND	ND	ND	ND	ND	ND
Diagnosis	0.91 (0.44–1.91)	0.812	ND	ND	ND	ND	ND	ND
PH (yes versus no)	1.41 (0.47–4.20)	0.538	ND	ND	ND	ND	ND	ND
Treatment								
Use PSL (mg/day)	1.00 (0.97–1.03)	0.920	ND	ND	ND	ND	ND	ND
O_2_ flow (L/min)	1.69 (1.01–2.62)	0.018	ND	ND	ND	ND	ND	ND
Physiologic								
FVC predicted (%)	0.94 (0.89–0.98)	0.003	ND	ND	ND	ND	ND	ND
FEV_1.0_ (%)	0.96 (0.93–1.00)	0.065	ND	ND	ND	ND	ND	ND
DLco predicted (%)	0.94 (0.90–0.98)	0.005	ND	ND	ND	ND	ND	ND
Δ N_2_ (%)	1.20 (0.93–1.56)	0.163	ND	ND	ND	ND	ND	ND
Resting SpO_2_ (%)	0.77 (0.55–1.06)	0.111	ND	ND	ND	ND	ND	ND
Lowest SpO_2_ (%)	0.93 (0.84–1.02)	0.126	ND	ND	ND	ND	ND	ND
Δ SpO_2_ (%)	1.06 (0.96–1.16)	0.270	ND	ND	ND	ND	ND	ND
SpO_2_ 1 minute (%)	0.91 (0.84–0.99)	0.029	ND	ND	ND	ND	ND	ND
Modifide Borg Scale	1.21 (0.98–2.18)	0.067	ND	ND	ND	ND	ND	ND
Laboratory data								
KL-6 (U/ml)	1.00 (1.00–1.01)	0.365	ND	ND	ND	ND	ND	ND
P/F ratio	0.99 (0.98–1.00)	0.073	ND	ND	ND	ND	ND	ND
Propensity score	ND	ND	2.90 (0.25–33.2)	0.392	1.13 (0.11–11.9)	0.92	0.09 (0.09–1.01)	0.51

Multivariable analysis indicates the adjusted effect by applying propensity score which is a conditional probability given by other clinicopathologic factors including age, sex, BMI, diagnosis, PH, use PSL, Supplemental O_2_ flow, FVC predicted, FEV_1.0_, DLco predicted,

Δ N_2_, Resting SpO_2_, Lowest SpO_2_, Δ SpO_2_, SpO_2_ 1 minute, Modifide Borg Scale, KL-6, P/F ratio. Definition of abbreviations: HR, hazard ratio; CI, indicates confidence interval; ND, not done; SpO_2_ recovery index; {(SpO_2_ 1 minute in to recovery after 6MWT minus lowest SpO_2_)/resting SpO_2_} × 100; HRR1, HR at the end of 6MWT minus HR after 1 minute at the end of 6MWT; BMI, body mass index; PH, Pulmonary hypertension; PSL, Prednisolone; O_2_ flow, Supplemental O_2_ flow; Δ SpO_2_, SpO_2_ at the end of the 6MWT minus SpO_2_ at baseline; SpO_2_ 1 minute, SpO_2_ 1 min into recovery after 6MWT; KL-6, Krebs von den Lungen-6; P/F ratio, PaO_2_/FIO_2_ ratio.

## Discussion

To the best of our knowledge, this is the first study to reveal a relationship between SpO_2_ recovery index values obtained from 6MWT and pulmonary diffusion capacities in patients with IP. It is also the first exploration of the predictors of rehospitalization predictors due to acute IP exacerbation and respiratory failure. SpO_2_ recovery index from 6MWT for IP inpatients was most strongly associated with pulmonary diffusion capacity and was a strong predictor for rehospitalization due to acute IP exacerbation and respiratory failure. When the 4% cutoff generated from ROC curves was used as a reference for SpO_2_ recovery index value for rehospitalization, the risk of rehospitalization during the follow-up period decreased by 0.3-fold with every 1% increase in SpO_2_ recovery index. Multivariate Cox regression analysis identified HRR1 obtained from 6MWT as a rehospitalization predictor for respiratory-related events, supporting previous studies on mortality. However, in this study, SpO_2_ recovery index was as a stronger predictor than HRR1.

Patients with IP with EIH have a poor prognosis, indicating the importance of evaluating EIH^5,27^. In clinical, radiological and physiological scoring systems for predicting prognosis in patients with IP, PaO_2_ during cardiopulmonary exercise testing is a significant survival rate predictor and the strongest prognosis predictor^5^. A recent American Thoracic Society consensus statement suggested a 4% decrease in saturation during exercise as an adverse prognostic sign in idiopathic pulmonary fibrosis (IPF)^1^. Many of these studies focused on desaturation/PaO_2_ during cardiopulmonary exercise testing. However, registry data suggest that cardiopulmonary exercise tests are rarely used to assess prognostic predictions in IPF patients^28^, possibly relating to the expense and limited availability of this diagnostic modality. 6MWT is a simple, convenient, inexpensive test requiring minimal medical personnel and can be performed in an office setting^29^. In this study, clinical data derived from 6MWT were analyzed. SpO_2_ recovery index from 6MWT was significantly associated with the gas exchange index %DLco, suggesting the estimation of pulmonary diffusion capacity levels using SpO_2_ recovery index after 6MWT without performing detailed pulmonary function tests (difficult to conduct due to insufficient facilities and environmental problems). These results may help in risk management during exercise therapy and determining oxygen levels during home oxygen therapy introduction.

For rehospitalization predictors, causes of deaths in patients with IP were not specifically identified in previous studies. HR changes during exercise and HRR after exercise, demonstrated in many previous studies, may reflect cardiac function and are presumed to be outcomes, a large part of which is accounted for by mortality attributable to cardiac disease as a complex. Previous studies included large patient numbers with high right ventricular systolic pressure (RSVP), using cardiac medication^14^, and who suffered PH after the study/had PH-related mortality^13^. To explain HR changes during and HRR changes after exercise as mortality predictors, Heindl^30^ noted abnormal sympathetic activation in patients with chronic respiratory failure, including some with pulmonary fibrosis. Most deaths occur from the progression of lung fibrosis rather than from commonly occurring comorbidities^15–17^. Frequent hospitalization for respiratory problems are common and associated with death^15–17^. Identifying rehospitalization predictors due to respiratory-related events (respiratory failure and acute IP exacerbation) was necessary. Unlike previous studies, our study limited the reasons for rehospitalization to IP exacerbation and respiratory-related events, including respiratory failure. Respiratory system function had a greater impact on the analysis and may justify why SpO_2_ recovery index after 6MWT, which is more relevant to the pulmonary diffusion capacity than HHR1, was a rehospitalization predictor for respiratory-related events. SpO_2_ recovery index was a useful index with clinical applicability regardless of whether the resting SpO_2_ level was high/low because it was adjusted with resting SpO_2_ for compensating oxygen consumption effect.

These results allow for a novel means of predicting respiratory-related events, such as acute IP exacerbation and respiratory failure and all-cause mortality, including that due to cardiac diseases. Evaluating SpO_2_ recovery index after 6MWT predicts rehospitalization due to acute IP exacerbation and respiratory failure, aiding in clinical practice.

Limitations of this study are that independent variables used in the multivariate analysis were limited because of the small sample size and the short, 1-year follow-up period, which can be addressed by subject accumulation and longer follow-up. This study excluded patients with immeasurable 6MWT, such as those using a high-flow-rate oxygenator and with a history of bone and joint diseases. Therefore, this method is not applicable to all patients in clinical practice and should be used for prognosis prediction after appropriate patient selection. SpO2 recovery is useful in predicting rehospitalization due to respiratory-related events. However, there is no clear intervention for minimizing rehospitalization due to respiratory-related events risk, and improvement of exercise tolerance and lower leg muscle strength by rehabilitation may be important, other than pulmonary diffusion capacity due to drugs.

## Conclusion

SpO_2_ recovery index after 6MWT in hospitalized patients with IP was most closely related to pulmonary diffusion capacity and the strongest independent rehospitalization predictor due to respiratory-related events. In clinical practice, pulmonary diffusion capacity can be estimated using SpO_2_ recovery indices obtained from 6MWT in appropriately selected patients and may help during exercise therapy. It was also useful in predicting rehospitalization due to respiratory-related events.
